# Perspectives on Player Performance during FIFA World Cup Qatar 2022: A Brief Report

**DOI:** 10.3390/sports11090174

**Published:** 2023-09-05

**Authors:** Luís Branquinho, Pedro Forte, Ronaldo V. Thomatieli-Santos, Elias de França, Daniel A. Marinho, José E. Teixeira, Ricardo Ferraz

**Affiliations:** 1 Department of Sports, Higher Institute of Educational Sciences of the Douro, 4560-708 Penafiel, Portugal; 2 Research Centre in Sports Sciences, Health Sciences and Human Development (CIDESD), 6201-001 Covilhã, Portugal; 3 Research Centre of Higher Institute of Educational Sciences, CI-ISCE/ISCE Douro, 2620-379 Ramada, Portugal; 4 Department of Sport Sciences and Physical Education, Polytechnic Institute of Bragança (IPB), 5300-253 Bragança, Portugal; 5 Department of Biosciences, Federal University of São Paulo, São Paulo 04040-003, Brazil; 6 Department of Sports Sciences, University of Beira Interior, 6201-001 Covilhã, Portugal

**Keywords:** match running performance, fixture congestion, weather

## Abstract

Changing the date of the FIFA World Cup Qatar 2022 may represent a factor to consider for the expected performance of participating players. This was due to fixture congestion at the start of the season and expected weather conditions during the competition. Thus, the main purpose of this brief report was to critically analyze the potential impact of changing the competition date and weather conditions on players’ performance. In addition, a brief description about the performance during the World Cup is also provided. For the research, the Web of Science, PubMed and SPORTDiscus databases were accessed using the primary keywords FIFA World Cup and World Soccer Cup associated with the secondary keywords match running performance, fixture congestion, fatigue and weather conditions. After applying inclusion and exclusion criteria, 52 articles were considered for analysis. The results seem to indicate that although changes were expected due to the modifications made (i.e., the competition date and scheduling congestion), the performance of the players seems not to have been affected in terms of the analyzed indicators. Furthermore, it seems possible to identify some patterns in the behavior of the teams that reached the most advanced stages of the competition.

## 1. Introduction

When it was confirmed that the FIFA World Cup 2022 would be held for the first time in a Middle Eastern country (Qatar) [1], a series of studies were carried out to mitigate the impact of high temperatures that are felt in this region of the globe [2,3,4], which can reach an average of 43 °C during the months of June and July [4]. Previous studies warned that there could be negative effects on the performance of soccer players when exposed to extreme weather conditions (i.e., high temperatures) [5,6,7,8]. For this reason, one of the measures taken was to change the usual date on which the FIFA World Cup takes place to the months of November and December when temperatures are relatively lower [3]. Even so, changing the competition date may have posed an additional challenge, particularly for teams that have players who played in the main European Leagues (i.e., the Premier League, La Liga, Bundesliga, Serie A and Ligue 1), given that the impact this change can have on players’ performance has not yet been clarified [9]. In fact, for the first time in history, a FIFA World Cup took place in the middle of the competitive season, which required that the coaches (the club and national teams) redoubled their attention in the management of training and game loads. This is due to the fact that the change in the date of the competition caused an increase in the physical and mental demands imposed on the players due to the increase in the number of games played [10] during the first phase of the season. Furthermore, to monitor fixture congestion impact, metrics related to match running performance have been used before [11,12,13], as they are also representative of the physiological demands imposed by the game [14,15,16]. Thus, the main objective of this review was to critically analyze the potential impact that the historic change in the usual competition date (June–July to November–December) and weather conditions had on the performance of players who played in the main European leagues and who participated in the FIFA World Cup 2022. In addition, a brief report on what happened during the competition will be provided.

## 2. Materials and Methods

### 2.1. Search Strategy

**Figure 1:**
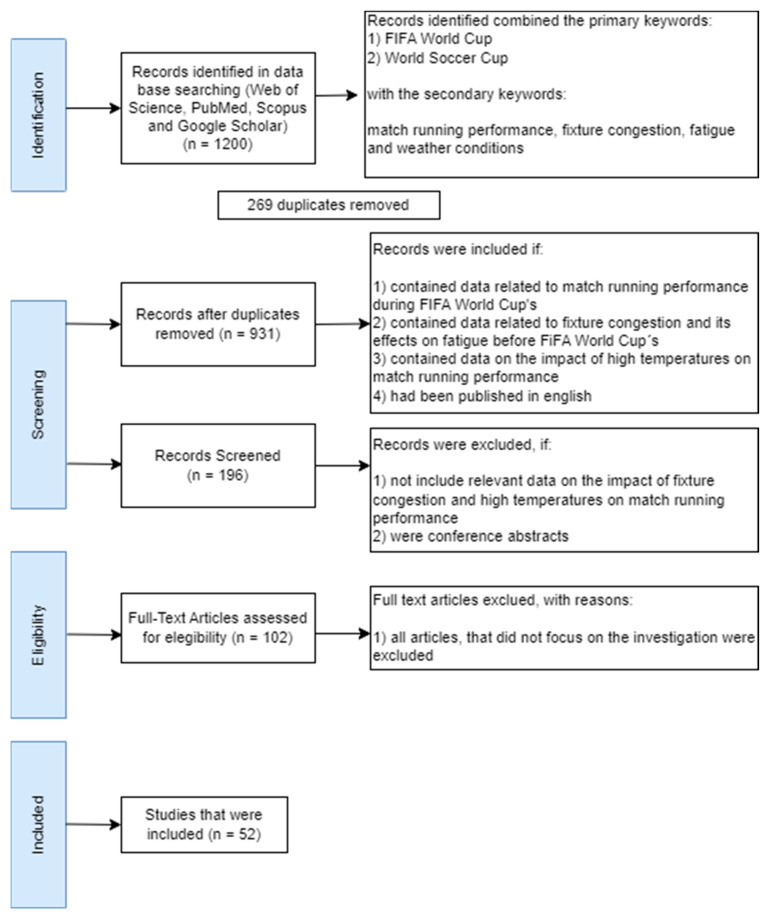
Flow diagram adapted from PRISMA 2009 [19].

To carry out this review, the available literature was investigated by searching the electronic databases Web of Science, PubMed and SPORTDiscus (between 1 September and 15 October 2022) for relevant publications (between 2000 and 2022), using the primary keywords “FIFA World Cup” and “World Soccer Cup” associated with the secondary keywords “match running performance”, “fixture congestion”, “fatigue” and “weather conditions”. Inclusion criteria for these articles were articles (1) containing data related to match running performance during the FIFA World Cups; (2) containing data related to fixture congestion and its effects on fatigue before the FIFA World Cups; (3) containing data on the impact of high temperatures on performance; and (4) published in English. Studies were excluded if (1) they did not include relevant data on the impact of fixture congestion and high temperatures on match running performance and (2) were conference abstracts (Figure 1). To assess the quality of the studies, a validated protocol was used [17].

In addition, the data used in this study were obtained from the official FIFA World Cup 2022 website: http://www.fifa.com/worldcup/archive/quatar2022/index.html, accessed on 30 March 2023. The dependent variables, distance covered and high speed distance, were measured for each of the participating teams. The values of the average distances covered (i.e., total and high speed) are presented in kilometers. Descriptive and inferential statistics were performed using SPSS (version 22; SPSS Inc., Chicago, IL, USA).

### 2.2. Methodological Quality

Table 1 shows the summary of the checklist present in the STROBE guidelines according to the recommendations previously provided [18]. Of the fifty-two studies included in the brief report, five studies scored between 14 and 15, six with 16, twenty-four with 17, nine with 18 and three with 19 points.

## 3. Results and Discussion

### 3.1. Factors That May Have Affected Performance in FIFA World Cup 2022

#### 3.1.1. Changes to the Regular Season Schedule

**Figure 2:**
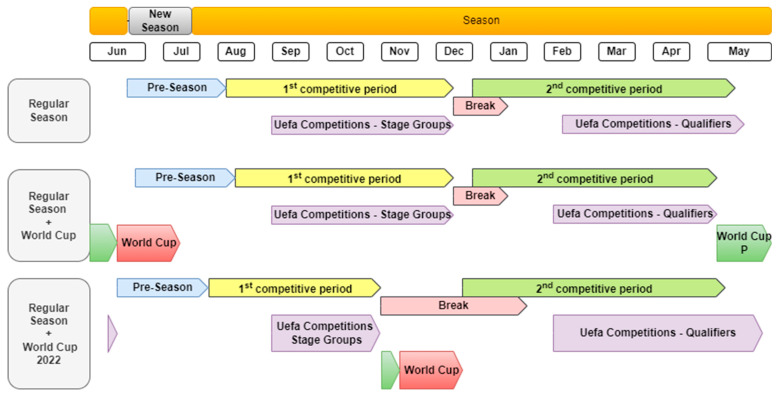
Season schedule in European championships with and without international tournaments.

Soccer is characterized as a multifaceted sport that requires players to be able to perform a series of complex technical actions, as well as a set of intermittent actions that involve different movement patterns, also requiring a constant tactical adjustment in response to the constraints of the game [20,21]. Over the last few years, due to technological evolution, there has been an exponential increase in studies that investigate technical [22], tactical [23,24] and physical [25] performances during a soccer game. Particularly regarding physical spectrum investigations, running performance has been one of the most investigated research topics in soccer [26,27], and tracking systems that allow a detailed quantification of the physical demands imposed have contributed to this [28,29]. In this regard, in particular, the total distance covered, in addition to being able to provide useful information on performance, also globally represents the physiological demands imposed during a match [14,15,16]. Previous investigations have reported that match running could be influenced by seasonal variations [30,31], and these factors could have gained prominence during the FIFA World Cup 2022. Even so, there seems to be no consensus in the literature on the topic, given that while some authors argue that there seems to be a trend of decline in running performance throughout the season due to accumulated fatigue [32], others state that the competitive phase does not seem to be determinant for the external load responses obtained [27]. In some of the main European championships, the regular season consists of a preseason period (i.e., 4–6 weeks) and two competitive periods (i.e., ±20 weeks each), with a winter break in some of them (e.g., Bundesliga) [33,34,35] (Figure 2).

Usually, during the years when European championships and World championships take place, there is a 4-to-5-week break between the end of domestic competitions and the start of international competitions [36,37]. However, at the FIFA World Cup 2022, the preparation period was considerably shorter, particularly in Europe, as there was only 1 week of pre-competition preparation after the interruption of the national championships, and the impact of this reduction needs to be clarified (Figure 2). This contrasts with what occurred in other areas of the world where the championships were interrupted earlier, allowing a longer preparatory period for the FIFA World Cup (15 days—North America and 30 days—Asia).

**Figure 3:**
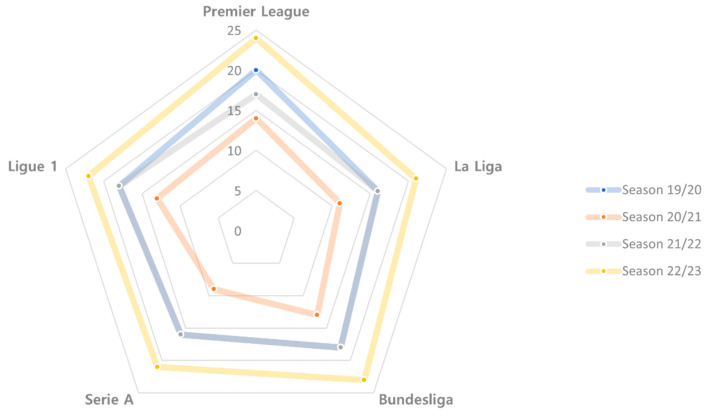
Matches played by teams that participated in all competitions over the last 4 seasons from the first matchday to the stoppage time for the FIFA world Cup 2022.

In addition, compared to the same competitive period of previous seasons, there was a greater concentration of games in the first 4 months of the season, and this was mainly reflected in the group stage of the UEFA competitions, which usually takes place over 3 months but was concentrated into just 8 weeks in the 2022/2023 season (Figure 2). Usually, the teams that supply more players to the national teams are also involved in more national and international competitions (i.e., domestic leagues, domestic cups, the UEFA Champions League, the UEFA Europe League and the UEFA Conference League), and compared to the same period in previous years, there was, on average, an increment of five to seven games (Figure 3). Fixture congestion periods are common in elite soccer; however, the accumulated external load can increase fatigue rates and condition performance and increase the risk of injury [38], and for these reasons, the impact of these changes needs to be clarified.

On the other hand, hosting the FIFA World Cup 2022 in this specific period (i.e., November and December) could have had a positive impact on player performance. This may be due to the fact that this competition took place at the end of the first competitive period, where there is usually already an accumulation of games due to the national cups usually played in this period [39]. Corroborating this evidence, a recent investigation reported positive differences in physical performance during the first competitive period compared to the second competitive period in professional soccer players [40].

Over the last few years, numerous investigations have shown that, over time, match running performance indicators have gradually increased in the FIFA World Cups from 1966 to 2018, which translates into an increase in the demands imposed by a soccer match [41,42,43,44,45,46,47]. Thus, the expectation is that in the FIFA World Cup 2022, this trend continued; however, the various structural changes in the competition (i.e., the changes in the season schedule) may have affected the performance of players, and this needs to be clarified. In addition, clubs look ahead to the fitness level at which their players will return to domestic competition. Undoubtedly, the FIFA World Cup 2022 schedule was surrounded by a series of uncertainties and doubts that could only be answered at the end of the tournament through new investigations and evidence. In addition, it would also be of extreme interest to verify whether the patterns that are found in this competition are in line with what occurs in other team sports whose international competitions take place during the competitive season (e.g., futsal, handball and roller hockey).

#### 3.1.2. Weather Conditions

During the summer, temperatures in Qatar fluctuate between 25 and 46 °C, which in combination with a relative humidity of up to 100%, can make you experience thermal sensations above 50 °C [4]. Extreme values of temperature are highly discouraged for humans under normal conditions, as well as during physical exercise, as the body temperature can rise up to 41.5 °C [48], interrupting the thermoregulatory response and compromising performance [49,50]. Fundamentally for these reasons, the decision was made to move the FIFA World Cup to a cooler time of year given (i.e., November and December) due to the potential impact this factor could have on player performance (i.e., match running performance). Previous evidence has reported changes in player performance in FIFA World Cup matches due to the combination of heat stress and explosive drills [51,52]. Corroborating this evidence, previous studies have reported that the ability of players to perform high-intensity exercise in environments with high temperatures is significantly reduced, which can translate negatively into sports performance (i.e., match running performance) [6,7,8]. The development of hyperthermia appears to decrease neuromuscular function with changes that occur at the peripheral and central levels and at these levels, can result in reduced voluntary muscle contraction and force production [53,54], which are fundamental for the decisive actions of the game. The organization of the tournament tried to mitigate the effects of the heat by implementing cooling systems that keep the indoor temperature of the stadiums at 27 °C, a bold and innovative measure that still may have limitations; therefore, future investigations are necessary [4]. Following this line of reasoning, it would be interesting for future investigations to investigate the potential impact that the inclusion of air conditioning systems in stadiums may have on the development of respiratory infections.

#### 3.1.3. Performance during FIFA World Cup 2022

**Figure 4:**
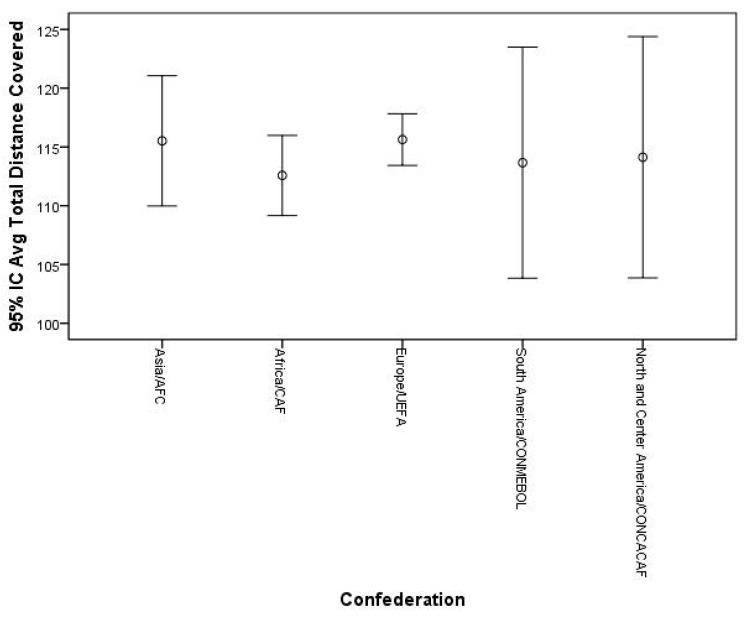
Descriptive statistics of total distance covered (km) by confederation.

**Figure 5:**
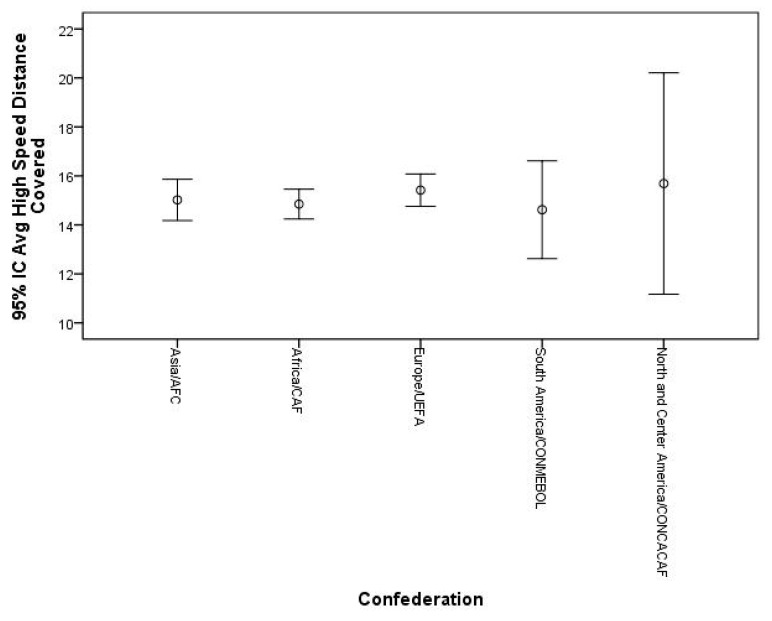
Descriptive statistics of high-speed distance covered by confederation.

**Figure 6:**
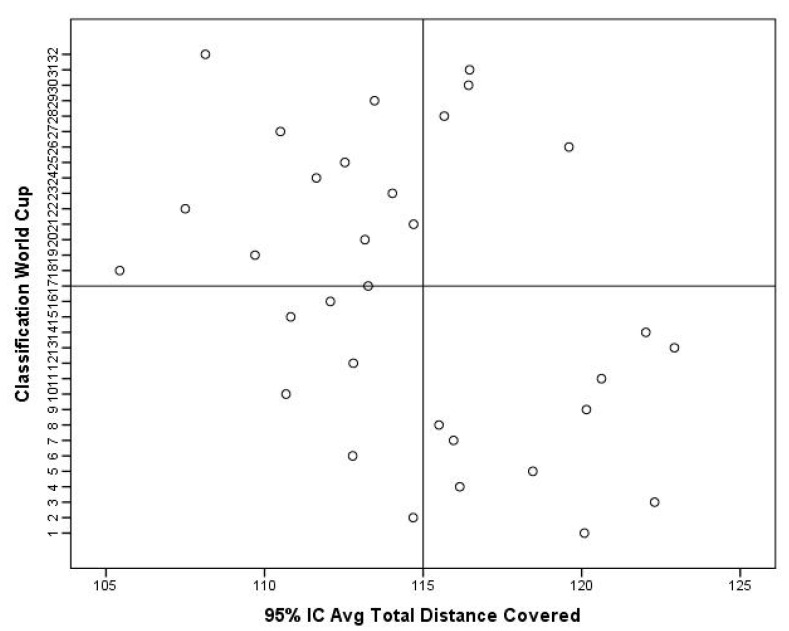
Descriptive statistics of total distance covered with respect to the classification obtained in the World Cup 2022.

**Figure 7:**
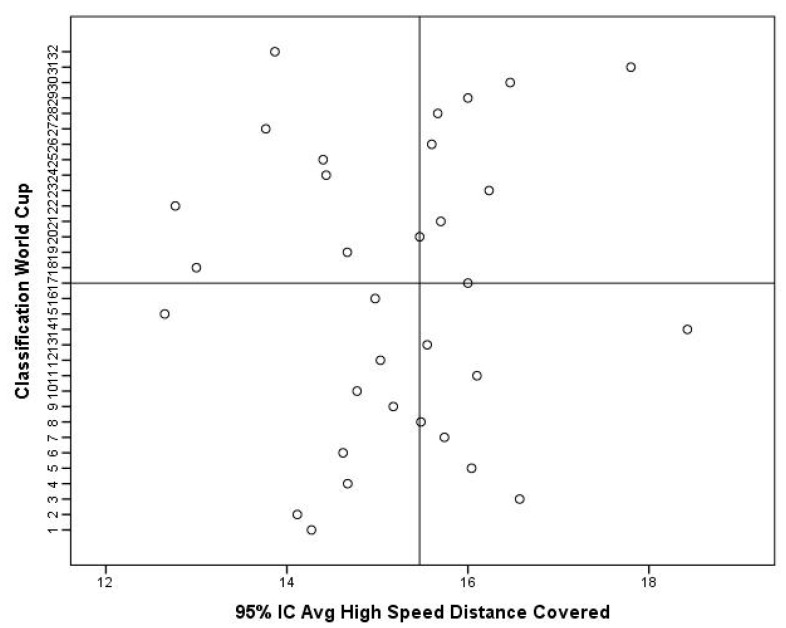
Descriptive statistics of high-speed distance covered with respect to the classification obtained in the World Cup 2022.

After hosting the FIFA World Cup 2022, some considerations can be made. Match statistics seem to indicate that there were no significant differences for external load indicators (i.e., the total distance covered and total distance covered at high intensity) between teams from different confederations (*p* ≤ 0.05). In other words, the competitive congestion found, particularly in Europe (where most of the players who reached the final stages played), seems not to have negatively influenced performance. In addition, the data show that teams from Europe were those with the highest metrics for the total distance covered and total distance covered at high intensity, with smaller variations among the other confederations (Figure 4 and Figure 5). In addition, there seems to be a tendency for teams that reached the most advanced stages of the competition to have greater total distances covered compared to other teams (Figure 6). Regarding the total high-speed distances covered, a curious phenomenon can be seen. In fact, there seems to be a tendency for the teams that got further in the competition to show lower values for this indicator (Figure 7). These data can derive from several factors (i.e., game strategy), as well as an effective pacing strategy (i.e., conscious effort regulation). Pacing strategies have been reported in the literature as effective mechanisms to manage effort in short-term competitions [12,55,56]. Apparently, even though they cover greater distances, the teams that go further run at lower intensities, which, in fact, can be an important factor as the competition advances. The data also allow us to infer that the weather conditions seem not to have negatively influenced the performance of the players given that the analyzed indicators are in line with what has been reported in football matches under normal weather conditions, having even been higher than those recorded in the World Cup of 2018 [57].

## 4. Conclusions

Until now, there were still many uncertainties about the potential impact that the changes that occurred in the FIFA World Cup 2022 schedule had on player performance, and in fact, these remain to be fully clarified. Although the literature seems to indicate that there could be changes in performance due to scheduling congestion and environmental conditions, among other factors, the analysis resulting from this work seems to indicate that the changes verified did not have a significant impact on the external load variables analyzed (i.e., the total distance covered and high-speed distance covered). Preliminarily, and based on the results found, it can be said that changing the date of the competition seems to have even had a positive impact on the performance of the players. These conclusions are based on the fact that there is an increase in the performance of the variables analyzed compared to the 2018 World Cup and the fact that it was the World Cup with the most goals ever. Even so, this study should be seen only as the starting point for deeper analyses, with a greater number of variables and indicators that can help clarify, in detail, the performance of all participating teams. In addition, it may still be interesting to investigate the potential differences that occurred between teams from different confederations to try to find different game patterns/behaviors. At a time when the championships are coming to an end, it may be essential to carry out analyses that can indicate the potential impact that the world championship had on the participating players (e.g., injuries, breaks in performance). The information resulting from these studies can not only create new perspectives on changes to the usual World Cup schedule but may also be useful in preparation for future competitions.

## Figures and Tables

**Table 1:** Strengthening the Reporting of Observational Studies in Epidemiology (STROBE) checklist for observational studies.

	1	2	3	4	5	6	7	8	9	10	11	12	13	14	15	16	17	18	19	20	21	22	Total
Sulayem et al. [1]	0	1	1	1	1	1	0	0	0	0	1	1	0	1	1	1	0	1	1	1	1	0	14
Zhong et al. [2]	0	1	0	1	1	1	1	1	0	0	1	1	1	0	0	1	1	1	1	1	1	0	15
Matzarakis et al. [3]	1	1	1	1	1	1	1	1	0	0	1	1	1	0	0	1	1	1	0	1	1	0	16
Sofotasiou et al. [4]	0	1	1	1	1	1	1	1	1	0	1	1	0	0	1	0	0	1	1	1	1	0	15
Özgünen et al. [5]	0	1	1	1	1	1	1	1	1	0	1	1	1	1	1	0	0	1	0	1	0	0	15
Mohr et al. [6]	0	1	1	1	1	1	1	1	1	0	1	1	1	1	1	1	0	1	0	1	0	1	17
Mohr et al. [7]	0	1	1	1	1	1	1	1	1	0	1	1	1	1	1	1	0	1	0	1	1	0	17
Grantham et al. [8]	0	1	1	1	1	1	1	1	1	0	1	1	0	1	1	0	0	1	0	1	0	0	14
Zouhal et al. [9]	0	1	1	1	1	1	1	1	0	0	1	1	1	1	1	0	0	1	0	1	0	0	14
Anderson et al. [10]	0	1	1	1	1	1	1	1	1	0	1	1	1	1	1	1	0	1	1	1	1	0	18
Vieira et al. [11]	0	1	1	1	1	1	1	1	1	0	1	1	1	1	1	1	0	1	1	1	0	1	18
Jones et al. [12]	0	1	1	1	1	1	1	1	1	0	1	1	1	1	1	1	0	1	1	1	0	0	17
Carling et al. [13]	0	1	1	1	1	1	1	1	0	0	1	1	0	0	1	1	0	1	1	1	0	0	14
Reilly et al. [14]	0	1	1	1	1	1	1	1	1	0	1	1	1	1	1	1	0	1	1	1	0	0	17
Castellano et al. [15]	0	1	1	1	1	1	1	1	1	0	1	1	1	1	1	1	0	1	1	1	0	0	17
Di Salvo et al. [16]	0	1	1	1	1	1	1	1	1	0	1	1	1	1	1	1	0	1	1	1	0	0	17
Banno et al. [19]	1	1	1	1	1	1	1	1	0	0	1	1	1	1	1	1	0	1	1	1	0	0	17
Yang et al. [20]	0	1	1	1	1	1	1	1	1	0	1	1	1	1	1	1	0	1	1	1	1	1	19
Jiménez-Reyes et al. [21]	0	1	1	1	1	1	1	1	1	1	1	1	1	1	1	1	0	1	1	1	0	0	18
Ade et al. [22]	0	1	1	1	1	1	1	1	0	0	1	1	1	1	1	1	0	1	0	1	1	1	17
Rein et al. [23]	0	1	1	1	1	1	1	1	1	0	1	1	1	1	1	0	0	1	0	1	0	0	15
Memmert et al. [24]	0	1	1	1	1	1	1	1	1	0	1	1	1	1	1	1	0	1	1	1	1	1	19
Dolci et al. [25]	1	1	1	1	1	1	1	1	0	0	1	1	1	1	1	0	0	1	1	1	0	1	17
Palucci et al. [26]	1	1	1	1	1	1	1	1	1	0	1	1	1	1	1	0	0	1	0	1	0	1	17
Teixeira et al. [27]	0	1	1	1	1	1	1	1	1	0	1	1	1	1	1	1	0	1	1	1	0	1	18
Branquinho et al. [28]	0	1	1	1	1	1	1	1	0	0	1	1	1	1	1	1	0	1	1	1	0	1	17
Branquinho et al. [29]	0	1	1	1	1	1	1	1	0	0	1	1	1	1	1	1	0	1	1	1	0	1	17
Ferraz et al. [30]	0	1	1	1	1	1	1	1	1	0	1	1	1	1	1	1	0	1	1	1	0	1	18
Padrón-Cabo [31]	0	1	1	1	1	1	1	1	1	0	1	1	1	1	1	1	0	1	1	1	0	0	17
Springham et al. [32]	0	1	1	1	1	1	1	1	0	0	1	1	1	1	1	1	0	1	1	1	0	0	16
Eliakim et al. [33]	1	1	1	1	1	1	1	1	1	0	1	1	1	1	1	1	0	1	1	1	0	0	18
Szymanski et al. [34]	0	1	1	1	1	1	1	1	1	0	1	1	1	1	1	0	0	1	0	1	0	0	15
Goossens et al. [35]	0	1	1	1	1	1	1	1	1	0	1	1	1	1	1	1	0	1	1	1	0	0	17
McCall et al. [36]	1	1	1	1	1	1	1	1	1	0	1	1	1	1	1	1	0	1	1	1	0	0	18
Bok et al. [37]	0	1	1	1	1	1	1	1	0	0	1	1	1	1	1	1	0	1	1	1	0	0	16
Dupont et al. [38]	0	1	1	1	1	1	1	1	1	0	1	1	1	1	1	1	0	1	0	1	0	1	17
Clemente et al. [39]	1	1	1	1	1	1	1	1	1	1	1	1	1	1	1	1	0	1	1	1	0	0	19
Silva [40]	0	1	1	1	1	1	1	1	1	0	1	1	1	1	1	1	0	1	1	1	0	0	17
Chmura et al. [41]	0	1	1	1	1	1	1	1	0	0	1	1	1	1	1	1	0	1	1	1	0	0	16
Dvorak et al. [42]	0	1	1	1	1	1	1	1	1	0	1	1	1	1	1	1	0	1	1	1	0	0	17
Clemente et al. [43]	0	1	1	1	1	1	1	1	1	0	1	1	1	1	1	1	0	1	1	1	0	1	18
Tuo et al. [44]	0	1	1	1	1	1	1	1	1	0	1	1	1	1	1	1	0	1	1	1	0	0	17
Rago et al. [45]	1	1	1	1	1	1	1	1	0	0	1	1	1	1	1	1	0	1	1	1	0	0	17
Silva et al. [46]	0	1	1	1	1	1	1	1	1	0	1	1	1	1	1	1	0	1	1	1	0	1	18
Wallace et al. [47]	0	1	1	1	1	1	1	1	1	0	1	1	1	1	1	1	0	1	1	1	0	0	17
Brotherhood [48]	0	1	1	1	1	1	1	1	0	0	1	1	1	1	1	1	0	1	1	1	0	0	16
Périard et al. [49]	0	1	1	1	1	1	1	1	0	0	1	1	1	1	1	1	1	1	1	1	0	0	17
Maughan et al. [50]	0	1	1	1	1	1	1	1	1	0	1	1	0	0	1	0	0	1	1	1	0	0	14
Nassis et al. [51]	0	1	1	1	1	1	1	1	1	0	1	1	1	1	1	1	0	1	1	1	0	0	17
Tschopp et al. [52]	0	1	1	1	1	1	1	1	0	0	1	1	1	1	1	1	0	1	1	1	0	0	16
Linnane et al. [53]	0	1	1	1	1	1	1	1	1	0	1	1	1	1	1	1	0	1	1	1	0	0	17
Girard et al. [54]	0	1	1	1	1	1	1	1	1	0	1	1	1	1	1	1	0	1	1	1	0	0	17

Note: 1: Title and abstract; 2: background/rationale; 3: objectives; 4: study design; 5: setting; 6: participants; 7: variables; 8: data sources/measurement; 9: bias; 10: study size; 11: quantitative variables; 12: statistical methods; 13: participants; 14: descriptive data; 15: outcome data; 16: main results; 17: other analyses; 18: key results; 19: limitations; 20: interpretation; 21: generalizability; 22: funding.

## Data Availability

http://www.fifa.com/worldcup/archive/quatar2022/index.html.
